# Accurate and Scalable Classification of Colonoscopy Neoplasia Using Machine Learning and Natural Language Processing

**DOI:** 10.14309/ctg.0000000000000959

**Published:** 2024-12-17

**Authors:** Brendan Broderick, Jason Greenwood, Douglas Mahoney, Kelli Burger, Sushil Kumar Garg, Michael B. Wallace, Suryakanth R. Gurudu, Derek Ebner, John Kisiel

**Affiliations:** 1 Center for the Science of Health Care Delivery, Mayo Clinic, Rochester, Minnesota, USA;; 2 Division of Family Medicine, Mayo Clinic, Rochester, Minnesota, USA;; 3 Division of Biomedical Statistics and Informatics, Mayo Clinic, Rochester, Minnesota, USA;; 4 Division of Gastroenterology and Hepatology, Mayo Clinic Health System, Eau Claire, Wisconsin, USA;; 5 Division of Gastroenterology and Hepatology, Mayo Clinic, Jacksonville, Florida, USA;; 6 Division of Gastroenterology and Hepatology, Mayo Clinic, Scottsdale, Arizona, USA;; 7 Division of Gastroenterology and Hepatology, Mayo Clinic, Rochester, Minnesota, USA.

**Keywords:** random forest, natural language processing, predictive modeling, neoplasia detection rate, pathology report analysis

## Abstract

**INTRODUCTION:**

Colorectal cancer remains a leading cause of cancer associated death in the United States and colonoscopy the primary screening strategy for prevention. Rates of adenomatous and serrated neoplasia detection are inversely associated with postcolonoscopy colorectal cancer. This crucial quality metric depends on accurate ascertainment of colorectal neoplasia findings from both endoscopy and histopathology records. We aimed to assess the feasibility of a random forest machine learning model to rapidly and accurately categorize colorectal neoplasia from electronic health record data.

**METHODS:**

A retrospective cohort study compared neoplasia detection rates among individuals undergoing colonoscopy at a large academic institution to develop a rule-based algorithm to categorize colorectal neoplasia from endoscopy reports and pathology systematized nomenclature of medicine – clinical terms. This cohort provided a large training set to develop a natural language processing system using a random forest approach to automatically classify unstructured pathology findings into adenoma, serrated, or advanced neoplasms. This system was manually validated through an independent holdout set.

**RESULTS:**

The training set comprised 35,953 unstructured pathology reports with matched systematized nomenclature of medicine – clinical terms from 95,188 unstructured colonoscopy reports. The final model was assessed on an independent holdout set of 337 manually annotated procedures obtaining an area under the receiver operating characteristic curve of 0.997 (confidence interval [CI] 0.994–1), 0.99 (CI 0.98–1), and 0.99 (CI 0.98–0.99) for prediction of adenoma, serrated, and advanced lesions, respectively.

**DISCUSSION:**

The random forest-based hybrid natural language processing system for classification of colonoscopy results was both accurate and explainable. NLP combined with effective machine learning algorithms can provide a scalable strategy for colonoscopy quality monitoring.

## Graphical abstract

**Figure:**
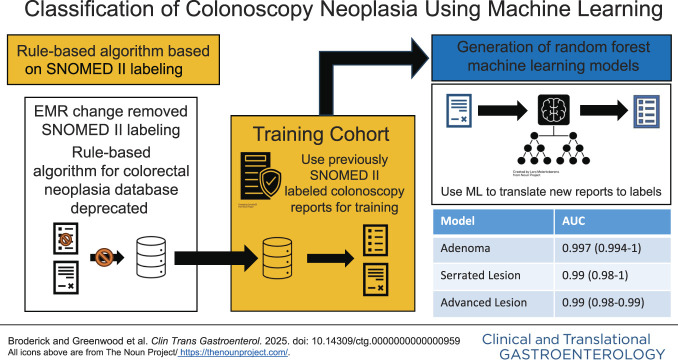

## INTRODUCTION

The American Cancer Society projects 154,270 new cases of colorectal cancer (CRC) and 52,900 associated deaths in 2025 alone (1). Colonoscopy is the most used CRC screening strategy in the United States and is often considered the gold standard (2). Colonoscopy is a critical tool for CRC screening, capable of both detecting and removing colorectal lesions at the time of screening or in follow-up of positive noninvasive CRC screening tests. Colonoscopy reports and matching histopathology records are further used to risk-stratify patients and determine the time for reassessment (3) and to measure the quality of the colonoscopy examination itself.

The adenoma detection rate (ADR) is the most important quality indicator in colonoscopy (4). Higher ADRs correlate with lower postcolonoscopy CRC and mortality (5). Historically, ADR was calculated by dividing the number of patients with at least one detected adenoma by the total number of colonoscopies conducted for average risk screening by an individual endoscopist. Recently however, the number of eligible indications for ADR monitoring has expanded and a new neoplasia benchmark has been created for serrated lesions (4). Multi-society task force recommendations prioritize these colonoscopy quality indicators and although the methodology for calculating neoplasia detection rates is well described, they do not provide implementation strategies (4). Separately, where implementation tips have been offered, the guidance is generic or reinforces the importance of updating established quality monitoring systems (6,7). There is a gap in guiding those that do not yet have a system for monitoring neoplasia detection rates. Considering the time, cost, and staffing needs to measure this quality indicator, it is not surprising that some practices do not calculate detection rates or calculate them incorrectly (8). Machine learning tools may provide an effective strategy for bridging this gap.

Generative artificial intelligence could provide a means for reporting neoplasia detection rates. However, the computational costs and potential for hallucinations are 2 key barriers to implementing these models (9). Admittedly, the risk for hallucinations is low for text classification of documents. A separate concern is the manner in which future large language model iterations may affect performance on models that were validated on an artificial intelligence platform that is being sunset and the high energy cost/carbon footprint/expense of generating these models (10). Fortunately, there are other predication models that do not have these limitations. Random forest ensemble learning uses supervised learning to accurately predict several classification outcomes (11). Random forest models depend on structured data elements, which can be generated through natural language processing (NLP). Although other NLP methods have demonstrated the ability to accurately categorize colonoscopy and pathology reports (12,13), random forest modeling has not yet been extensively evaluated in classifying colorectal neoplasia.

We aimed to develop and validate an automated tool that leverages NLP techniques to breakdown text of pathology reports into single word or word combination tokens, which then serve as data elements for random forest machine learning to predict neoplasia status. We sought to provide a scalable and efficient approach to pathology classification thereby enabling reporting of neoplasia detection rates.

## METHODS

### Data set Analysis

After Mayo Clinic Institutional Review Board approval, we conducted a retrospective cohort study to characterize and quantify colorectal neoplasia among patients that had undergone colonoscopy across the Mayo Clinic enterprise (Rochester, MN; Scottsdale, AZ; Jacksonville, FL; and the associated Midwest community-based centers (Mayo Clinic Health System).

Earlier work performed by our group provided the training cohort for this study (14). In brief, colonoscopy procedures from the period of October 1, 2014 to January 1, 2017 contained across multiple electronic health record (EHR) vendors including Cerner and General Electronic were evaluated to generate a historic rule-based algorithm to identify colorectal neoplasia. Colonoscopy procedures were queried using the institutional endoscopy report database (ProVation; Wolters Kluwer Health, Minneapolis, MN) to capture polyp size and colonoscopy quality end points such as bowel preparation and maximal extent reached. During that study period, pathology reporting used systematized nomenclature of medicine – clinical term labeling. Systematized nomenclature of medicine – clinical terms include morphological descriptors to categorize findings such as adenomas, serrated, and more advanced lesions (e.g., adenocarcinoma). Thus, the coding standardization during that time-period provided a rich set of unstructured reports to match with structured data and labeling to develop an expansive training cohort for this study. We used the colonoscopy procedure paired with an unstructured pathology report to create a training Data set. The systematized nomenclature of medicine – clinical terms served as our training set labels and only text from the pathology report served as our training features.

Importantly, our institution began conversion to the Epic (Madison, WI) EHR in early 2017, at which point in time pathology discontinued reporting with systematized nomenclature of medicine – clinical terms.

### Modeling-high level design

Using random forest, we developed 3 models of the unstructured findings to create a score for 3 key categories: adenoma, sessile serrated lesion (sessile serrated adenoma/polyp and/or traditional serrated adenoma; hyperplastic polyps were excluded), or advanced lesion (CRC cancer, polyp ≥10 mm of any histology [including hyperplastic], villous histology, or high-grade dysplasia). Based on thresholds for each of these models (see below), pathology report text were classified into the 3 key categories above or marked as negative findings if all 3 models were negative. We used R (R Foundation for Statistical Computing, Vienna, Austria) to preprocess text with the tokenizer package, leveraging the tidymodels metapackage for machine learning tasks. Within tidymodels, rsample handled training/test and cross-validation splitting, whereas parsnip and tune were used for model specification, hyperparameter tuning, and fitting (15). This pipeline facilitated a reproducible and systematic approach for data preparation and final model evaluation.

### Tokenization

We use 3 types of tokenization for our modeling purposes, tokenization of single words, and n-grams (n = 2, and n = 3).

Unigram (1-gram): Single words

1. Example: “adenoma detected” → [“adenoma,” “detected”]

Bigram (2-gram): 2-word combinations

1. Example: “adenoma detected” → [“adenoma detected”]

Trigram (3-gram): 3-word combinations

1. Example: “sessile serrated polyp” → [“sessile serrated polyp”]

We took the most common occurrence of 100 unigrams, 200 bigrams, and 300 trigrams from the data to produce a Data set that has 500 structured features. We chose to use only 500 features in an effort to maximize predictive yield and not over fit the models which can be commonplace in Data sets with high dimensionality. If the token existed in the note, the feature was the number of times that the n-gram occurs in the note.

Example

Example Pathology Note Entry

**Table:** 

Note_id = 100
A. Colon, sigmoid, polyp, endoscopic biopsy: The specimens examined contained both adenoma and sessile serrated polyp. Multiple levels examined

Example Model Output Table

The example output table represents the data elements that the random forest model can use as possible features, these are 6 examples of 500 total features used.

**Table:** 

note_id	sessile	sessile.serrated	sessile.serrated.polyp	adenoma	polyp	adenoma.polyp
100	1	1	1	1	1	0

### Evaluation

**Figure 1.**
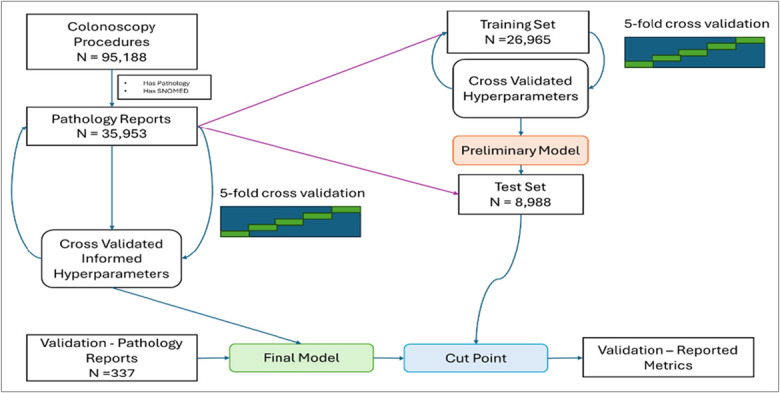
Study flow diagram. A large training cohort was divided into a training and test set. The final prediction model was validated through a manually annotated hold outset.

We split our training Data set into a training set of 26,965 and test set of 8,988 (Figure 1). For each of the 3 models (adenoma, serrated, and advanced lesion) we took the training set to determine the most common n-grams across notes using the tokenization method discussed previously and used the result to narrow feature selection. We then performed 5-fold cross-validation on the training set to decide on the best hyperparameter set to be used. For the random forest model, we considered 3 different hyperparameters minimum n in node size, number of predictors sampled at each tree, and number of total trees. Note that by tuning minimum n in node size, we let the tree depth vary until minimum n in node size is reached. The hyperparameter ranges considered by random forest were:

1. Minimum number of datapoints in node—2,3,4,5
2. Number of predictors randomly sampled—1,2,3,4, and 5
3. Number of trees—500 to 2,000 by 200

The best performing hyperparameter set from the cross-validation based on the area under the receiver operating characteristic curve (AUC) is applied to the training. The AUC for each of the 3 models in the test set serves as an unbiased estimate of out-of-sample performance of the final model.

### Cut point

Based on the performance metrics, sensitivity, specificity, positive predictive value, and negative predictive value of the test set, we choose cut points for classification for adenoma, serrated, and advanced lesions that struck the best balance of performance. The test set was used to determine cut point because performance metrics were unbiased estimates of true performance and had higher chance of being replicated out of our original sample. We opted for an empirically determined cut point by visually examining the score distribution alongside our chosen metrics to decide on cut points that achieved well in all metrics, rather than relying on a pure algorithmic optimization approach. The Youden index agreed with our chosen cut points as our chosen values were 96th percentile (adenoma), 99th percentile (serrated), and 98th percentile (advanced) in the index.

### Final model

Although we used nested k-fold cross-validation to evaluate model performance and report metrics such as sensitivity, specificity, F1, and AUC on the test set, a final model was trained on the full Data set before being locked prior to validation. To use the full information of the training Data set and minimize over-fitting, we report the only the hyperparameter set and performance metrics of the final tested model, which will be used to make predictions in future work.

### Data set Validation

To provide a true independent holdout set, we reserved a sample of colonoscopy reports from our structured endoscopy database (ProVation; Wolters Kluwer Health) paired with unstructured pathology reports from March 1, 2017 to June 1, 2024, post new EHR deployment. This convenience sample consisted of cases that had been reviewed by a clinician (D.W.E.) experienced in performing colonoscopies and reviewing associated pathology reports. The clinician manually annotated each case into one of the same 3 predicted categories: adenoma, serrated, and advanced lesion, based on established pathological and polyp size criteria. This manually obtained categorization was then compared with the model's prediction to assess the model's performance–sensitivity, specificity, F1 and AUC.

## RESULTS

### Data set Analysis

The analysis Data set comprised 35,953 unstructured pathology reports with matched systematized nomenclature of medicine – clinical terms from 95,188 unstructured colonoscopy reports; a breakdown of the labels for the report can be found in Supplementary Digital Content (see Supplementary Table 1, http://links.lww.com/CTG/B440). In addition, demographics and counts for each histology label are provided in Supplementary Digital Content (see Supplementary Table 2, http://links.lww.com/CTG/B440). The test set (N = 8,988) had an AUC and confidence interval (CI) of 0.997 (CI 0.996–0.998), 0.999 (CI 0.998–1), and 0.998 (CI 0.997–1) for the adenoma, serrated, and advanced lesion models, respectively. This signaled that the model performance on the test set had a very high degree of accuracy for predicting histology from the pathology report.

### Final model

The model hyperparameters were optimized based on the 5-fold cross validation of the full set maximizing for AUC for each of the model categories as mentioned previously (Table 1). Further details about the ranges of AUC are provided in Supplementary Digital Content (see Supplementary Table 9, http://links.lww.com/CTG/B440).

**Table 1.** Hyperparameters of final model

Model	Min no. of datapoints in node	No. of predictors randomly sampled	No. of trees
Adenoma	2	5	1,000
Serrated lesion	4	5	2,000
Advanced lesion	2	5	2,000

### Validation

The validation set was performed through analysis of the locked final model performance on the hold out of 337 manually annotated procedures (Table 2). Neoplasia categorization and patient demographics are provided in Supplementary Digital Content (see Supplementary Table 2, http://links.lww.com/CTG/B440). The cases were reviewed when the predicated categorization of neoplasia did not match that from manual review and the rational for miscategorization was explainable. For example, a large polyp had been removed during colonoscopy, and the pathology report detailed a moderately differentiated adenocarcinoma arising in a tubular adenoma with high-grade dysplasia. The model predicted adenoma and advanced neoplasia, however, expert review only qualified advanced neoplasia. Another example included a colonoscopy hosted in combination with an esophagogastroduodenoscopy. During esophagogastroduodenoscopy a biopsy of a gastric polyp (fundic gland polyp) was miscategorized as a colonic adenoma. Although the sample is limited in the validation set (17 combination procedures) the AUCs were 0.94 (CI 0.84–1), 1.00 (CI 1.00–1.00), and 0.95 (CI 0.87–1.00) in the adenoma, sessile, and advanced models, respectively.

**Table 2.** Validation set performance of models

Model	Sensitivity (CI)	Specificity (CI)	F1 (CI)	AUC (CI)
Adenoma	0.99 (0.94–0.99)	0.96 (0.94–0.98)	0.97 (0.94–0.99)	0.997 (0.994–1)
Serrated lesion	0.96 (0.86–0.99)	1.0 (0.98–1)	0.95 (0.89–0.99)	0.99 (0.98–1)
Advanced lesion	1.0 (0.94–1)	0.98 (0.95–0.99)	0.94 (0.89–0.98)	0.99 (0.98–0.99)

CI, confidence interval.

The performance was highly accurate, showing the ability to classify pathology and colonoscopy reports based on the tokenization of the note illustrating it as a powerful and simplistic NLP method. Additional model metrics and the confusion matrix for each outcome can be found in Supplementary Digital Content (see Supplementary Tables 3–7, http://links.lww.com/CTG/B440s).

## DISCUSSION

We developed a random forest machine learning model system for classification of patients' colonoscopy results (procedure and pathology) to label for any of the 3 categories: adenoma, serrated, or advanced lesion. The model was to supplement the previous coding performed with systematized nomenclature of medicine – clinical terms across multiple EHR platforms that were lost due to an EHR unification upgrade–leaving us with only unstructured pathology reports. With access to the previous systematized nomenclature of medicine – clinical terms data, which were paired with similar unstructured pathology reports, this provided an opportunity to use these as labels for the training and test set. Model selection was evaluated at the beginning of the process as small language models and large language models were becoming more popular and cheaper to use. However, our institution at the time had institutional review board and information technology restrictions to cloud-based large language model usage that came with increased cost to develop/maintain and delays in approval for research. Ultimately, we pursued our hybrid model and designed it to run on as little as a laptop to take up a small memory/computation footprint and intern run with minimal cost for our information technology/Gastroenterology Department as compared with production ready commercial large language models that require large cloud instances, large amounts of memory, and customized hardware. This would also enable deployment by both small practices and large institutions running the model in parallel to the EHR using common import/exporting features (e.g., Fast Healthcare Interoperability Resources) or embedding within EHRs (where supported) with little information technology effort. In a post hoc analysis we generated a confusion matrix of a key word/regular expression search strategy. Although there was good performance for predicting serrated lesions, the prediction for adenomas was less accurate when compared with our random forest modeling (see Supplementary Table 10, http://links.lww.com/CTG/B440). Ultimately, random forest machine learning seems to be accurate and scalable for classification of colorectal neoplasia.

The models were able to perform well, having an AUC for all 3 predictions at 0.99. To our knowledge, our model represents the first system to use a hybrid of NLP and random forest modeling for categorizing colonoscopy findings. However, this technique of random forest machine learning algorithms is not new to the field of gastroenterology. This tool has been used in a number of ways such as to predict Crohn's disease relapse (15,16), classify hepatic steatosis/fibrosis (17), and even predict future colon polyps (18). Although there is similar accuracy for histology extraction through NLP alone, incorporation of random forests enables a clear advantage in the ability to delineate/explain what factors lead to the model's prediction, which is not always possible through NLP systems. Although not incorporated into the current model, random forest techniques can also use other types of data that are beyond the capabilities of NLP alone. Realizing this random forest-based hybrid NLP system has a very quick processing time and is not expensive to run, it has the potential to enable frequent and timely reporting of colonoscopy indicators for proceduralists. This has important clinical implications as regular feedback on colonoscopy performance has been found to improve ADR overtime (19).

The models developed have some limitations related to scope, data structure, and report outputs. Regarding scope, the current iteration is most accurate for colonoscopy alone and a paired pathology report. An event of miscategorization was observed during a combination procedure, as described under the validation results. This limitation can readily be addressed through additional model training but was beyond the scope for this study. Regarding data structure and report outputs, we have identified limitations that are shared with NLP models. For example, there is concern surrounding generalizability. The accuracy of the model when applied to an outside data set is unknown, theoretically, the complex decision trees from our model should be able to better categorize colonoscopy findings compared with NLP alone. However, how items are stored and reported vary by endoscopy database vendor and by institution. We would have to modify inputs, validate the model, and potentially retrain the model realizing free text reports will vary from institution to institution even when following pathology reporting standard guidelines. These are like other studies as many are rule based or rely on certain structures/wording (15). We have explored model performance at our institution among a cohort of patients with inflammatory bowel disease realizing these individuals were excluded in the generation of the model. Interestingly, the model does seem to maintain accuracy. We acknowledge that other institutions may not have used systematized nomenclature of medicine – clinical terms which provided a labeled data set for our training. For those that may consider replicating our model, they would need to consider manual annotation of the pathology reports for labeling a training set and could consider using systematized nomenclature of medicine – clinical terms methodology for this labeling (see Supplementary Table 1, http://links.lww.com/CTG/B440). Any efforts at generating an automated program for categorization of neoplasia detection rates should consist of a team with appropriate content expertise (i.e., data scientist and gastroenterologist).

Although our primary aim was to explore the feasibility of a random forests machine learning model to accurately classify neoplasia detected during colonoscopy, it does have immediate capabilities to report adenoma and serrated lesion detection rates. However, the current model does not classify all eligible hyperplastic lesions within the serrated lesion category. Strictly speaking, the definition for the sessile serrated lesion detection rate includes hyperplastic polyps greater than 5 mm proximal to the sigmoid colon (4,20). We elected to exclude hyperplastic polyps in our model unless the colonoscopy identified the polyp size as 10 mm or greater. In this event, the model would predict advanced neoplasia (considering the polyp size) but if only hyperplastic histology was found, a serrated lesion would not be categorized. Our justification for otherwise excluding hyperplastic polyps for the current iteration of the model is best demonstrated when considering polypectomy from the descending and sigmoid colon. At our center, 2 small polyps from these segments of the colon would be combined into one specimen jar. If histology is reported as an adenoma and hyperplastic polyp, it would be very arbitrary if the hyperplastic histology counts for the descending colon polypectomy rather than the sigmoid location. To avoid gaming in the reporting of detection rates, we excluded hyperplastic histology. The model may underestimate the detection of serrated lesions by under reporting small, proximal hyperplastic polyps.

Future work will focus on extracting more granular features of the colonoscopy report and pathology report—e.g., bowel preparation and location-based polyp information. Realizing reporting formats and database structures will evolve over time, our group is exploring a less ridged model so that model accuracy is preserved despite changes in reporting format. This would also allow the model to be used by outside institutions.

Overall, this study demonstrates the effectiveness of simple tokenization of text combined with effective machine learning algorithms for prediction of colonoscopy findings. In an independent expert reviewed hold out set, we achieved AUCs of above 0.99 in adenoma, serrated lesions, and advanced lesion detection. These findings highlight the ability to calculate neoplasia detection rates over a large EHR with ease and will be integral to answer many research related questions surrounding colonoscopy performance in the future.

## CONFLICTS OF INTEREST

**Guarantor of the article:** Derek Ebner, MD.

**Specific author contributions:** B.B.: data curation, investigation, writing—original draft, review and editing, lead. J.G.: data curation, investigation, writing—original draft, review and editing, lead. D.W.M.: formal analysis, methodology, validation. Writing—review and editing, supporting. K.N.B.: data curation, formal analysis, project administration, writing—review and editing, supporting. S.K.G.: review and editing, supporting. M.B.W.: writing—review and editing, supporting. S.R.G.: writing—review and editing, supporting. D.W.E.: conceptualization, funding acquisition, data curation, investigation, validation writing—review & editing, supporting. J.B.K.: conceptualization, funding acquisition, methodology, supervision writing—review and editing, supporting.

**Financial support:** This work was supported by a grant from the Kern Center for the Science of Health Care Delivery to D.W.E. and the National Cancer Institute (CA214679) to J.B.K.

**Potential competing interests:** None to report.

**Study Highlights**

**WHAT IS KNOWN**

✓ Colorectal neoplasia diagnosis rates are key quality metrics for colonoscopy performance.
✓ Calculation of colorectal neoplasia diagnosis rates is laborious requiring manual abstraction or automated tooling, which can be expensive.

**WHAT IS NEW HERE**

✓ Natural language processing and a random forest model provide a hybrid system that can accurately classify colorectal neoplasia, a requirement for detection rate estimation.
✓ This type of model requires a small footprint, is transparent, and runs with minimal cost, providing a scalable option for colonoscopy quality monitoring.

## Supplementary Material

## ABBREVIATIONS:

ADR: adenoma detection rate
AUC: area under the receiver operating characteristic curve
CI: confidence interval
CRC: colorectal cancer
EGD: esophagogastroduodenoscopy
EHR: electronic health record
NLP: natural language processing
NPV: negative predictive value
PPV: positive predictive value
