# Supplementary material for: Accurate and Scalable Classification of Colonoscopy Neoplasia Using Machine Learning and Natural Language Processing
Source: Clin Transl Gastroenterol. 2024 Dec 17;17(2):e00959. doi: 10.14309/ctg.0000000000000959 (PMC12922929; doi:10.14309/ctg.0000000000000959)
Supplement: Supplementary file 1 [file ct9-17-e00959-s001.docx]

**Supplement Table 1:**

SNOMED II code classification of training data. We curated classifications for morphology codes into three categories 1) adenomas 2) serrated lesions and 3) advanced lesions. If a patient’s pathology report had any of the codes in each of the categories, they were given a label of 1 in the training set, otherwise they were given a label of 0 or thought to be negative for the histology.

| Adenoma | M74000:Dysplasia, nos  M74001:Dysplasia, focal  M74003:~M74003  M74005:Dysplasia, atypical  M74006:Dysplasia, mild  M74007:Dysplasia, moderate  M74008:Dysplasia, severe  M76800:Polyp, nos  M76900:Polyposis, nos  M80000:Neoplasm, benign  M80001:Neoplasm, uncertain whether benign or malignant  M80002:Neoplasm, benign, in-situ  M80003:Neoplasm, malignant  M80006:Neoplasm, metastatic  M80009:neoplasm malignant uncertain whether  M80011:Tumor cells, uncertain whether benign or malignant  M80013:Tumor cells, malignant  M80102:Carcinoma in-situ, nos  M80103:Carcinoma, nos  M801031:Carcinoma, well differentiated  M801033:Carcinoma, nos, poorly differentiated  M80106:Carcinoma, metastatic, nos  M80109:Carcinomatosis  M81402:Adenocarcinoma in-situ  M81403:Adenocarcinoma, nos  M82102:Adenomatous polyp, nos, in-situ  M82103:Adenocarcinoma in adenomatous polyp  M821032:Adenocarcinoma in adenomatous polyp, mod. diff.  M82113:Tubular adenocarcinoma  M82200:adenomatous polyposis coli  M82210:multiple adenomatous polyps  M82612:adenocarcinoma in situ in villous adenoma  M82613:Adenocarcinoma in villous adenoma  M82632:Tubulovillous adenoma, in-situ  M82633 Tubulovillous adenoma, malignant  M800013:Neoplasm, uncertain whether benign or malignant, poorly differentiated  M81400:Adenoma, nos  M82100:Adenomatous polyp, nos  M82110:Tubular adenoma, nos  M82611:Villous adenoma, nos  M82630:Tubulovillous adenoma |
| --- | --- |
| Serrated Lesions | M76801:polyp, sessile  M82133:Serrated adenocarcinoma  M82130:Serrated adenoma |
| Advanced Lesions | M74008 Dysplasia, severe  M80003 Neoplasm, malignant  M80006 Neoplasm, metastatic  M80013 Tumor cells, malignant  M80102 Carcinoma in-situ, nos  M80103 Carcinoma, nos  M801031 Carcinoma, well differentiated  M801033 Carcinoma, nos, poorly differentiated  M80106 Carcinoma, metastatic, nos  M80109 Carcinomatosis  M81402 Adenocarcinoma in-situ  M81403 Adenocarcinoma, nos  M82103 Adenocarcinoma in adenomatous polyp  M821032 Adenocarcinoma in adenomatous polyp, mod. diff.  M82113 Tubular adenocarcinoma  M82612 adenocarcinoma in situ in villous adenoma  M82613 Adenocarcinoma in villous adenoma  M82632 Tubulovillous adenoma, in-situ  M82633 Tubulovillous adenoma, malignant  M82611 Villous adenoma, nos  M82630 Tubulovillous adenoma |

**Supplement Table 2:**

Analysis (Train/Test) and Validation data demographics and neoplasia.

|  | Analysis (N=35953) | Test (N=8988) | Training (N=26965) | Validation (N=337) |
| --- | --- | --- | --- | --- |
| **Age** |  |  |  |  |
| <55 | 12307 (34.2%) | 3156 (35.1%) | 9151 (33.9%) | 63 (18.7%) |
| 55-64 | 8955 (24.9%) | 2217 (24.7%) | 6738 (25.0%) | 67 (19.9%) |
| 65-74 | 9200 (25.6%) | 2299 (25.6%) | 6901 (25.6%) | 131 (38.9%) |
| >=75 | 5491 (15.3%) | 1316 (14.6%) | 4175 (15.5%) | 76 (22.6%) |
| **Gender** |  |  |  |  |
| Male | 19298 (53.7%) | 4846 (53.9%) | 14452 (53.6%) | 146 (43.3%) |
| Female | 16654 (46.3%) | 4142 (46.1%) | 12512 (46.4%) | 190 (56.4%) |
| Unknown | 1 (0.0%) | 0 (0.0%) | 1 (0.0%) | 1 (0.3%) |
| **Race** |  |  |  |  |
| White | 33269 (92.5%) | 8333 (92.7%) | 24936 (92.5%) | 320 (95.0%) |
| Asian or Pacific Islander | 686 (1.9%) | 160 (1.8%) | 526 (2.0%) | 9 (2.7%) |
| Black | 596 (1.7%) | 164 (1.8%) | 432 (1.6%) | 5 (1.5%) |
| American Indian/Alaska Native | 201 (0.6%) | 46 (0.5%) | 155 (0.6%) | 0 (0.0%) |
| Other | 1201 (3.3%) | 285 (3.2%) | 916 (3.4%) | 3 (0.9%) |
| **Ethnicity** |  |  |  |  |
| Not Hispanic or Latino | 33602 (93.5%) | 8384 (93.3%) | 25218 (93.5%) | 323 (95.8%) |
| Hispanic or Latino | 1198 (3.3%) | 294 (3.3%) | 904 (3.4%) | 8 (2.4%) |
| Unknown | 1153 (3.2%) | 310 (3.4%) | 843 (3.1%) | 6 (1.8%) |
| **Adenoma Polyps** |  |  |  |  |
| 0 | 19530 (54.3%) | 4913 (54.7%) | 14617 (54.2%) | 238 (70.6%) |
| 1 | 16423 (45.7%) | 4075 (45.3%) | 12348 (45.8%) | 99 (29.4%) |
| **Sessile Serrated Polyps** |  |  |  |  |
| 0 | 31228 (86.9%) | 7839 (87.2%) | 23389 (86.7%) | 291 (86.4%) |
| 1 | 4725 (13.1%) | 1149 (12.8%) | 3576 (13.3%) | 46 (13.6%) |
| **Advanced Lesion** |  |  |  |  |
| 0 | 34128 (94.9%) | 8542 (95.0%) | 25586 (94.9%) | 281 (83.4%) |
| 1 | 1825 (5.1%) | 446 (5.0%) | 1379 (5.1%) | 56 (16.6%) |

**Supplement Table 3: Adenoma Model Metrics**

| Sensitivity | Specificity | F1 | AUC |
| --- | --- | --- | --- |
| 0.99 (0.94 - 0.99) | 0.96 (0.94 - 0.98) | 0.97 (0.94 - 0.99) | 0.997 (0.994 - 1) |

**Supplement Table 4: Adenoma Confusion Matrix**

|  | Actual | | |
| --- | --- | --- | --- |
| Predicted |  | No Adenoma | Adenoma |
|  | No Adenoma | 230 | 1 |
|  | Adenoma | 8 | 98 |

**Supplement Table 5: Serrated Model Metrics**

| Sensitivity | Specificity | F1 | AUC |
| --- | --- | --- | --- |
| 0.96 (0.86 - 0.99) | 1.0 (0.98 - 1) | 0.95 (0.89 - 0.99) | 0.99 (0.98-1) |

**Supplement Table 6: Serrated Confusion Matrix**

|  | Actual | | |
| --- | --- | --- | --- |
| Predicted |  | No Serrated | Serrated |
|  | No Serrated | 289 | 2 |
|  | Serrated | 0 | 46 |

**Supplement Table 7: Advanced Lesion Model Metrics**

| Sensitivity | Specificity | F1 | AUC |
| --- | --- | --- | --- |
| 1.0 (0.94 -1) | 0.98(0.95 - 0.99) | 0.94(0.89 -0.98) | 0.99 (0.98-0.99) |

**Supplement Table 8: Advanced Lesion Confusion Matrix**

|  | Actual | | | |
| --- | --- | --- | --- | --- |
| Predicted |  | No Advanced lesion | Advanced lesion |  |
|  | No Advanced lesion | 273 | 0 |  |
|  | Advanced lesion | 6 | 58 |  |

**Supplement Table 9: AUC ranges with different hyperparameter**

| Model | Hyperparameters | AUC |
| --- | --- | --- |
| Adenoma - Minimum | Min number of datapoints in node: 2, Number of predictors randomly sampled: 1, Number of trees: 500 | 0.961 |
| Adenoma - Maximum | Min number of datapoints in node: 2, Number of predictors randomly sampled: 5, Number of trees: 1000 | 0.997 |
| Sessile- Minimum | Min number of datapoints in node: 1, Number of predictors randomly sampled: 3, Number of trees: 500 | 0.996 |
| Sessile- Maximum | Min number of datapoints in node: 4, Number of predictors randomly sampled: 5, Number of trees: 200 | 0.999 |
| Advanced- Minimum | Min number of datapoints in node: 1, Number of predictors randomly sampled: 3, Number of trees: 500 | 0.992 |
| Advanced- Maximum | Min number of datapoints in node: 2, Number of predictors randomly sampled: 5, Number of trees: 2000 | 0.999 |

**Supplement Table 10: Confusion Matrix of Key Word/Regular Expression search**

**Adenoma**

| Sensitivity | Specificity |
| --- | --- |
| 0.98 (0.94 - 0.99 | 0.92 (0.88 - 0.95 |

|  | Actual | | |
| --- | --- | --- | --- |
| Predicted |  | No Adenoma | Adenoma |
|  | No Adenoma | 221 | 1 |
|  | Adenoma | 17 | 98 |

**Sessile Serrated**

| Sensitivity | Specificity |
| --- | --- |
| 0.98 (0.89 - 0.99 | 1.0 (0.98 - 1.0 |

|  | Actual | | |
| --- | --- | --- | --- |
| Predicted |  | No Adenoma | Adenoma |
|  | No Adenoma | 289 | 2 |
|  | Adenoma | 0 | 47 |
